# A nomogram to predict lymph node metastasis risk for early esophageal squamous cell carcinoma

**DOI:** 10.1186/s12885-021-08077-z

**Published:** 2021-04-20

**Authors:** Xiaofeng Duan, Xiaobin Shang, Jie Yue, Zhao Ma, Chuangui Chen, Peng Tang, Hongjing Jiang, Zhentao Yu

**Affiliations:** Department of Esophageal Surgery, Tianjin Medical University Cancer Hospital and Institute, Key Laboratory of Cancer Prevention and Therapy, National Clinical Research Center for Cancer, Tianjin, 300060 China

**Keywords:** Esophagus, Cancer, Lymph nodes, Metastasis, Nomogram model

## Abstract

**Background:**

A nomogram was developed to predict lymph node metastasis (LNM) for patients with early-stage esophageal squamous cell carcinoma (ESCC).

**Methods:**

We used the clinical data of ESCC patients with pathological T1 stage disease who underwent surgery from January 2011 to June 2018 to develop a nomogram model. Multivariable logistic regression was used to confirm the risk factors for variable selection. The risk of LNM was stratified based on the nomogram model. The nomogram was validated by an independent cohort which included early ESCC patients underwent esophagectomy between July 2018 and December 2019.

**Results:**

Of the 223 patients, 36 (16.1%) patients had LNM. The following three variables were confirmed as LNM risk factors and were included in the nomogram model: tumor differentiation (odds ratio [OR] = 3.776, 95% confidence interval [CI] 1.515–9.360, *p* = 0.004), depth of tumor invasion (OR = 3.124, 95% CI 1.146–8.511, *p* = 0.026), and tumor size (OR = 2.420, 95% CI 1.070–5.473, *p* = 0.034). The C-index was 0.810 (95% CI 0.742–0.895) in the derivation cohort (223 patients) and 0.830 (95% CI 0.763–0.902) in the validation cohort (80 patients).

**Conclusions:**

A validated nomogram can predict the risk of LNM via risk stratification. It could be used to assist in the decision-making process to determine which patients should undergo esophagectomy and for which patients with a low risk of LNM, curative endoscopic resection would be sufficient.

## Background

Esophageal squamous cell carcinoma (SCC) is the most common pathological type in China [1]. Surgery after neoadjuvant therapy is the main treatment method with curative potential for early- or advanced-stage disease. Although the incidence of mortality and complication after esophageal surgery have decreased significantly in some high-volume cancer centers, esophagectomy can seriously affect the quality of life. To maintain the integrity of the esophagus and reduce the morbidity and mortality of the procedure, endoscopic resection has gradually become the treatment option for some patients with early-stage esophageal cancer [2]. Endoscopic resection, however, may not be curative and has several issues, including tumor residue; without lymph node dissection, it can lead to local recurrence and tumor spread. For patients with lymph node metastasis (LNM), radical lymph node dissection is crucial to decrease disease recurrence and prolong survival.

However, the incidence of LNM in patients with early-stage esophageal cancer is relatively high, especially for submucosal infiltrating tumors [3–8]. The LNM rate in T1a stage esophageal SCC (ESCC) patients is 0–15.4% and that in T1b stage ESCC patients is 15–51% [3–11]. Clinical staging currently relies on endoscopic ultrasound and positron emission tomography (PET) computed tomography (CT), but the limitations of these techniques do not allow for preoperative accurate detection of all LNMs [12–14]. In the JCOG0502 study, LNM occurred in 27% early-stage cases, as determined by pathological examinations [15]. Therefore, it is crucial to identify the risk factors of LNM for early esophageal cancer to aid in the decision-making process of the surgical procedure and treatment strategy. In this study, a nomogram model was developed and validated by another cohort to predict LNM and determine the surgical management strategy based on risk stratification in patients with early-stage ESCC.

## Methods

### Database

**Fig. 1:**
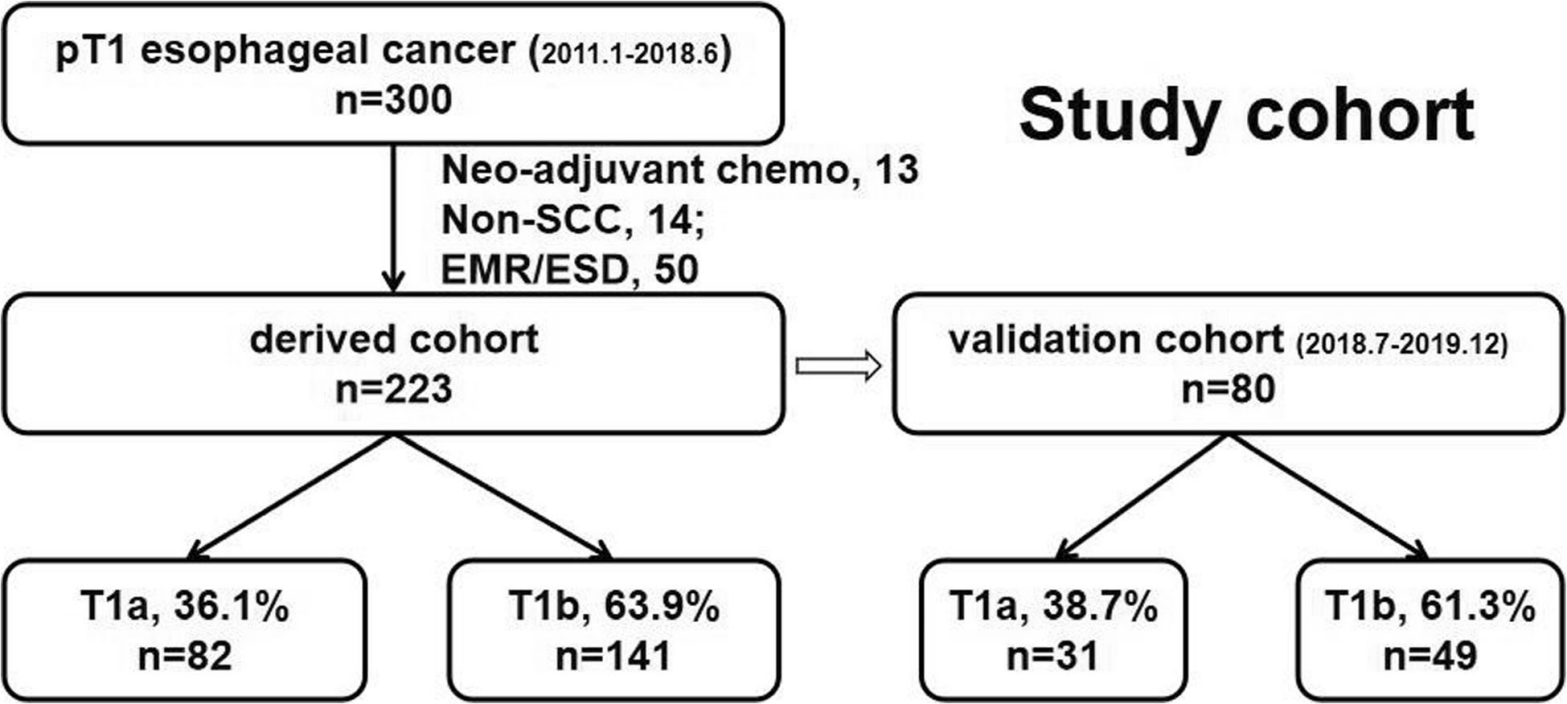
Study flow chart

Data on consecutive esophageal cancer patients at our cancer center between January 2011 and June 2018 were obtained from our retrospective database. Histopathology confirmed that 300 patients had pT1 stage esophageal cancer. Patients who underwent neoadjuvant therapy (*n* = 13), those with non-SCC (*n* = 14), and those who underwent primary endoscopic resection (*n* = 50) were excluded. Finally, a total of 223 patients were included in the present study, including 82 pT1a and 141 pT1b patients. The study flow chart is shown in Fig. 1. These patients were included as the derived cohort.

Another case cohort, later used as the validation cohort, included 80 patients who underwent esophagectomy between July 2018 and December 2019. Patients with the same inclusion and exclusion criteria as those in the derived cohort at the same cancer center were also identified.

Our study cohort was classified according to the seventh edition of the AJCC/UICC TNM classification of malignant tumors [16].

### Preoperative evaluation

Preoperative evaluation included physical examination, laboratory analysis, imaging examination and endoscopy. The diagnosis and staging of esophageal cancer were performed using endoscopic biopsy and endosonography. Ultrasound and CT combined with enhanced scanning determined local growth, lymph node status, and distant metastasis. In some cases, PET-CT was used to exclude metastatic diseases and evaluate tumor resectability.

### Surgical procedure

All patients included in this study underwent transthoracic esophagectomy with two- or three-field lymphadenectomy. For patients with upper esophageal cancer or suspicions LNM on preoperative examination, three-field lymphadenectomy was performed. For patients with middle/lower esophageal cancer and without suspicions LNM, two-field lymphadenectomy was performed. Most patients underwent minimally invasive esophagectomy (video/robot-assisted Ivor-Lewis or McKeown procedures). Open surgery was performed for some patients. The detailed surgical procedure could be found in our previous study [10].

### Statistical analysis

We used the statistical software package R (version 3.0.0, R Foundation for statistical calculations) and SPSS Statistics (version 18.0, SPSS Inc., Chicago, IL) for statistical analysis. Univariate analysis was performed using the χ2 test or Fisher’s exact test. Binary logistic regression analysis was used to investigate the LNM risk factors. Clinicopathological variables with a *P* value of < 0.01 in univariate analysis were included in the logistic regression analysis (two-sided and *P* < 0.05 was considered significant). In this study, the receiver operating characteristic (ROC) curve and Youden index were used to determine the optimal cutoff point for the continuous variables in the LNM prediction [17].

To test the LNM prediction ability for each risk factor and for comprehensive analysis, a ROC curve was constructed and the area under the curve (AUC) was considered an estimation of the prediction accuracy [17]. A nomogram model was constructed based on the parameters related to LNM in multivariate analysis.

## Results

### Patients’ characteristics

A total of 223 patients were used as the derivation cohort. The demographic and clinical characteristics of the patients are presented in Table 1. Overall, 36 patients had LNM, and the LNM rate was 16.1%. Of the 36 patients with LNM, 30 had pN1 disease, six had pN2 disease, and none had pN3 disease. The detailed LNM information is shown in Table 2.

**Table 1:** The characteristics of the patients with pT1 ESCC

Variables	Derivation, *n* = 223 (%)	Validation, *n* = 80 (%)
Sex, male/female	189/34	69/11
Age: median (range)	59 (42–77)	62 (42–75)
Location		
Upper	13 (5.8)	7 (8.8)
Middle	112 (50.2)	49 (61.3)
Lower	98 (44.0)	24 (30)
Tumor number		
Single	200 (89.7)	71 (88.8)
Mutifocal	23 (10.3)	9 (11.3)
Tumor size, cm: median (range)	2.0 cm (0.3–7.0 cm)	2.0 cm (0.6–8.0 cm)
Harvested lymph nodes: median (range)	19 (0–85)	19 (3–76)
Surgery		
Sweet	5 (2.2)	0
Ivor-Lewis	52 (23.3)	3 (3.75)
Mckeown	21 (9.4)	2 (2.5)
Minimally invasively esophagectomy	145 (65.0)	75 (93.7)
cT stage		
cT1a	65 (29.1)	37 (46.3)
cT1b	20 (9.0)	21 (26.3)
cT2	6 (2.7)	6 (7.5)
cT3	7 (3.1)	3 (3.8)
Unknown	125 (56.1)	13 (16.3)
Differentiation		
High-middle	188 (84.3)	65 (81.3)
Low	35 (15.7)	15 (18.8)
Depth of invasion		
Mucosal epithelial	34 (15.2)	8 (10)
Lamina propria mucosa	4 (1.3)	3 (3.8)
Muscularis mucosae	44 (19.7)	20 (25)
Submucosal	141 (63.7)	49 (61.3)
Lymphovascular invasion		
Not reported	207 (92.8)	77 (96.3)
Reported	16 (7.2)	3 (3.75)
Lymph node metastasis		
N0	187 (83.9)	67 (83.9)
N1	30 (13.5)	9 (13.5)
N2	6 (2.7)	3 (2.7)
N3	0 (0)	1 (1.25)

**Table 2:** The mapping of LNM according to tumor location

Station name			Location^a^	
	total	Upper (1)	Middle (*n* = 17) Lower (*n* = 18)	
Cervical				
104	1			1
Thoracic				
105	2			2
106recR	13	1	7	5
106recL	6	1	5	
108	3		1	2
107	1			1
109 L	1		1	
110	1		1	
Abdominal				
1	2		1	1
2	5			5
3	4		2	2
7	8			8
8	1			1

^a^ Based on 7th TNM stage system

### LNM risk factors

Univariate and multivariate logistic regression analysis were performed to identify the risk factors significantly associated with LNM (Table 3). Three variables were confirmed as independent LNM risk factors: tumor differentiation (odds ratio [OR] = 3.776, 95% confidence interval [CI] 1.515–9.360, *p* = 0.004), depth of tumor invasion (OR = 3.124, 95% CI 1.146–8.511, *p* = 0.026), and tumor size (OR = 2.420, 95% CI 1.070–5.473, *p* = 0.034).

**Table 3:** Risk factors for lymph node metastasis in deveriation cohort

Variables	LNM		*P* value	Logistic regression analysis		*P* value
	Negative (%) Positive (%)			OR	95%CI	
Sex			0.411	–	–	–
Male	156 (82.5)	33 (17.5)				
Female	30 (88.2)	4 (11.8)				
Age			0.418	–	–	–
< 60 years	92 (81.4)	21 (18.6)				
> = 60 years	94 (85.5)	16 (14.5)				
Tumor location			0.475	–	–	–
Upper	12 (92.3)	1 (7.7)				
Middle	96 (85.7)	16 (14.3)				
Lower	77 (80.2)	19 (19.8)				
Tumor number			0.075	–	–	–
Single	170 (85.0)	30 (15.0)				
Mutifocal	16 (72.7)	6 (27.3)				
Tumor size			0.029	2.420	1.070–5.473	0.034
< 2.5 cm	121 (87.7)	17 (12.3)				
> = 2.5 cm	65 (76.5)	20 (23.5)				
Differentiation			0.001	3.766	1.515–9.360	0.004
High-middle	164 (87.2)	24 (12.8)				
Low	22 (62.9)	13 (37.1)				
Depth of invasion			0.005	3.124	1.146–8.511	0.026
T1a	76 (92.7)	6 (7.3)				
T1b	111 (78.7)	30 (21.3)				
Lymphovascular invasion			0.001	–	–	–
Not reported	178 (86.0)	29 (14.0)				
Reported	8 (50.0)	8 (50.0)				

### Nomogram model

**Fig. 2:**
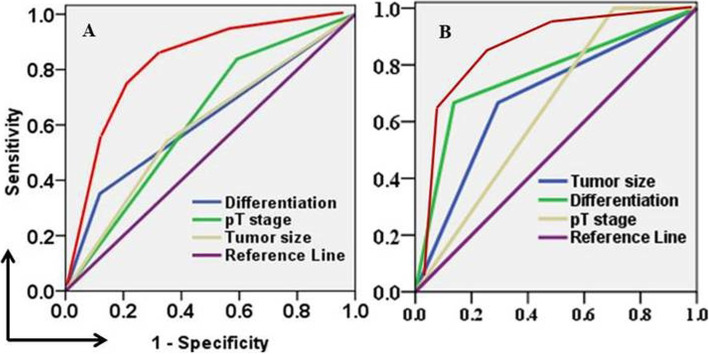
The prediction ability of each risk factor and the combined model. **a**. Derivation cohort. Tumor differentiation in blue line: AUC 0.617, 95% CI 0.509–0.724 (Sensitivity 0.356, Specificity 0.870); pT stage in green line: 0.623, 95% CI 0.532–0.714 (Sensitivity 0.812, Specificity 0.413); Tumor size in brown line: 0.596, 95% CI 0.494–0.697 (Sensitivity 0.552, Specificity 0.645); Combined full model in red line: 0.810 95% CI 0.742–0.895 (Sensitivity 0.821, Specificity 0.652). **b**. Validation cohort. Tumor differentiation in green line: AUC 0.765, 95% CI 0.596–0.933 (Sensitivity 0.650, Specificity 0.830); pT stage in brown line: 0.647, 95% CI 0.498–0.796 (Sensitivity 0.980, Specificity 0.312); Tumor size in blue line: 0.686, 95% CI 0.515–0.858 (Sensitivity 0.650, Specificity 0.682); Combined full model in red line: 0.830, 95% CI 0.763–0.902 (Sensitivity 0.820, Specificity 0.760)

We built a nomogram that incorporated LNM risk factors confirmed by logistic regression analysis. The prediction ability of the three risk factors were: tumor differentiation (0.617, 95% CI 0.509–0.724), depth of tumor invasion (0.623, 95% CI 0.532–0.714), and tumor size (0.596, 95% CI 0.494–0.697). The C-index was 0.810 (95% CI 0.742–0.895) (Fig. 2a).

**Fig. 3:**
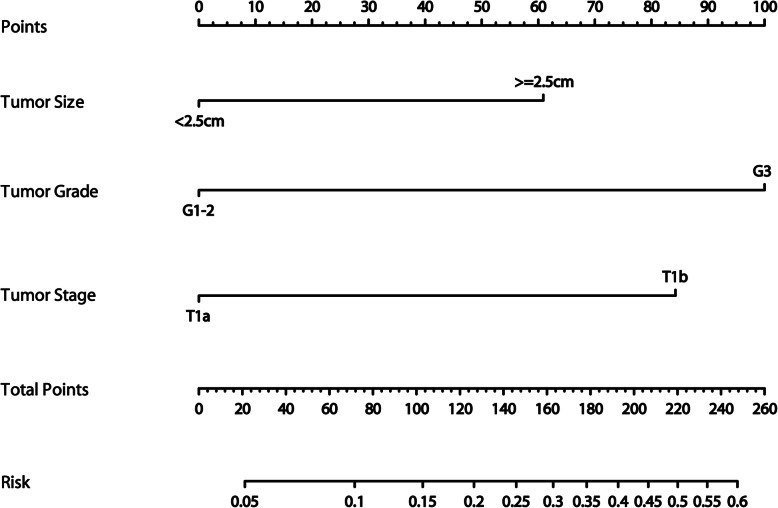
Nomogram to predict the LNM risk for each patient. The patient’s nomogram calculated the risk of LNM as 59%. Nomogram calculations are as follows: size ≥2.5 cm, which corresponds to 61 points; low differentiation, which corresponds to 100 points; pT1b, which corresponds to 85 points; this equals 246 total points, corresponding to a LNM rate of 59%

This compiled nomogram allowed for the estimation of the risk of LNM for each patient. It also illustrated the contribution of each risk factor to the overall LNM risk. The clinical use of the nomogram is illustrated in Fig. 3.

### Validation cohort

A total of 80 patients were used as the validation cohort. The demographic and clinical characteristics of the patients are summarized in Table 1**.** A total of 13 patients had LNM, and the LNM rate was 16.3%.

Consistent with the derivation cohort, tumor differentiation (OR = 3.902, 95% CI 1.843–10.354, *p* = 0.005), depth of tumor invasion (OR = 3.234, 95% CI 1.230–8.543, *p* = 0.010), and tumor size (OR = 2.674, 95% CI 1.876–6.320, *p* = 0.008) were confirmed as independent risk factors associated with LNM (Table 4). The C-index was 0.830 (95% CI 0.763–0.902) (Fig. 2b).

**Table 4:** Risk factors for lymph node metastasis in validation cohort

Variables	LNM		*P* value	Logistic regression analysis	*P* value
	Negative (%) Positive (%)			OR 95%CI	
Sex			1.000	–	–
Male	58 (84.1)	11 (15.9)			
Female	9 (81.8)	2 (18.2)			
Age			0.083	–	–
< 60 years	24 (75.0)	8 (25.0)			
> = 60 years	43 (89.6)	5 (10.4)			
Tumor location			0.131	–	–
Upper	6 (85.7)	1 (14.3)			
Middle	38 (77.6)	11 (22.2)			
Lower	23 (95.8)	1 (4.2)			
Tumor number			0.450	–	–
Single	60 (84.5)	11 (15.5)			
Mutifocal	7 (77.8)	2 (22.2)			
Tumor size			0.014	2.674 1.876–6.320	0.008
< 2.5 cm	46 (85.2)	4 (14.8)			
> = 2.5 cm	21 (80.8)	9 (19.2)			
Differentiation			0.008	3.902 1.843–10.354	0.005
High-middle	58 (89.2)	7 (11.8)			
Low	9 (60.0)	6 (40.0)			
Depth of invasion			0.012	3.234 1.230–8.543	0.010
T1a	30 (96.8)	1 (3.2)			
T1b	37 (75.5)	12 (24.5)			

### LNM risk stratification

Based on our nomogram model we constructed, the risk of LNM was stratified into different intensities starting from zero for cases with well-differentiated T1a-stage tumors measuring < 2.5 cm to the highest risk value of 59% for those with poorly differentiated T1b-stage tumors measuring > 2.5 cm (Table 5). The risk of LNM was stratified as low risk (< 10%), moderate risk (10–30%), and high risk (> 30%) based on the nomogram model.

**Table 5:** Risk categories based on LNM risk model

Risk categories	Risk stratification	LNM risk
Low risk	T1a, High/middle differentiated, < 2.5 cm	0
Low risk	T1a, High/middle differentiated, > = 2.5 cm	8%
Moderate risk	T1a, Low differentiated, < 2.5 cm	14%
Moderate risk	T1a, Low differentiated, > = 2.5 cm	28%
Moderate risk	T1b, High/middle differentiated, < 2.5 cm	12%
Moderate risk	T1b, High/middle differentiated, > = 2.5 cm	24%
High risk	T1b, Low differemtiated, < 2.5 cm	36%
High risk	T1b, Low differentiated, > = 2.5 cm	59%

## Discussion

With the increasing application of endoscopic resection in T1-stage esophageal cancer, the ability to reliably predict nodal disease has become especially important to select the best treatment modality. In this study, a nomogram model was developed to predict LNM in patients with pT1-stage ESCC. Our nomogram model incorporated three clinicopathological factors, including tumor invasion depth, tumor differentiation, and tumor size, which were significantly associated with LNM. Based on the nomogram, a surgical treatment strategy was established according to the LNM risk stratification.

The depth of tumor invasion is probably the most important factor influencing LNM. The LNM rate of ESCC in T1a stage is 0–15.4% [3–11]. In our series, the incidence of LNM in T1a patients was 7.3%, consistent with previous results. It is generally believed that the LNM rate of T1b ESCC is much higher than that of T1a ESCC, ranging from 15 to 51% [3–11]. Most previous studies have reported an incidence of about 20%, similar to the LNM rate of 22% for T1b tumors in our study. Some studies have divided the submucosa into three layers: sm1, sm2, and sm3 [18]. Nentiwch et al. [3] showed that tumors in all three layers had a higher and similar incidence of LNM [19]. However, Akutsu et al. [4] revealed that the metastasis and relapse rate increased with infiltration depth, at 16, 35, and 62% for tumors in sm1, sm2, and sm3, respectively. The current study also confirmed tissue differentiation as an independent risk factor, which had been revealed by many previous reports [2, 5, 6, 8]. The risk of LNM increased with that of increasing tumor size [2. 5]. The best cutoff value for tumor size was revealed as 2.5 cm in this study.

The current study also confirmed cervical, thoracic, and abdominal LNM in pT1-stage ESCC with bidirectional and skipping metastasis along the esophagus [15, 20, 21]. The most common LNMs were located at the recurrent laryngeal nerve and the left gastric artery [4, 22, 23]. The extent of lymph dissection in surgery should respect the LNM topography, even in early-stage ESCC.

Thus far, a few researchers have developed nomogram models to predict the risk of LNM in esophageal cancer [6, 11, 24–26]. For example, Zheng et al. [11] recently developed a nomogram for the prediction of LNM in early-stage ESCC patients. They included four items, namely depth of tumor invasion, tumor differentiation, tumor size, and lymphovascular invasion (LVI), and also validated the model with another case cohort. The rate of LVI in our study was 7.2% lower than that of 12.8% in a previous study [11] and another study of 20.5% [5]. The low reporting rate may be the reason why LVI was not confirmed as an independent prediction factor in our study. An external validation was also performed using an independent case cohort from our cancer center.

Based on the nomogram model we constructed, LNM risk was stratified into different intensities starting from zero, for cases with well-differentiated T1a-stage tumors less than 2.5 cm in size, to the highest risk value, at 59%, for those with poorly differentiated T1b-stage tumors bigger than 2.5 cm in size. The nomogram model can be used to help clinical decision-making to determine when endoscopic resection is adequate, or surgery is needed. In early-stage ESCC patients with metastatic nodes in preoperative evaluation, the standard treatment involves neoadjuvant therapy followed by esophagectomy. In patients without metastatic nodes, esophagectomy or endoscopic resection should be chosen as the primary treatment, according to the risk stratification. If sufficient stratification information cannot be obtained, endoscopic resection should be considered as the first treatment option, allowing a subsequent decision on whether additional esophagectomy is needed. Clinical decisions also need to consider other clinical factors, including patient age, physical condition, willingness, socioeconomic factors, and the level of comprehensive knowledge of the surgeon or endoscopy physician.

The limitations of this study must be considered. First, the retrospective single institution study obviously may be accompanied by the potential risks of patient selection bias. Second, most patients underwent two-field lymphadenectomy without cervical lymph dissection. Third, the reported LVI rate is lower than that reported in the literature, which may have led to its exclusion as a risk factor in the nomogram model. Moreover, some studies have classified T1b tumors into three layers (sm1, sm2, and sm3). However, detailed data on classification were not obtained in the present study. In this group, insufficient lymph node dissection was performed in a few patients, which may underestimate the frequency of LNM.

## Conclusions

The incidence of LNM in patients with early-stage ESCC is high. With the increasing application of endoscopic resection, it is especially important to reliably predict nodal disease to select the best treatment modality. Based on a group of pT1-stage ESCC patients, a nomogram was developed to predict LNM risk. Overall, LNM risk is higher in T1b than in T1a tumors. However, patients with T1a tumors who exhibited poor differentiation or size > 2.5 cm had a higher LNM rate than those with T1b tumors without these other high-risk factors. This nomogram may be used to assist surgeons in deciding which patients should undergo esophagectomy and to select those patients with low LNM risk and curative endoscopic resection is sufficient.

## Data Availability

The data generated during the current study are not publicly available since they will contain patient data and the informed consent agreement does not include sharing data publicly. An anonymized form of the data could be made available from the corresponding author upon reasonable request.

## Abbreviations

LNM: Lymph node metastasis
ESCC: Esophageal squamous cell carcinoma
OR: Odd ratio
CI: Confidence interval
